# Targeted Hsp70 fluorescence molecular endoscopy detects dysplasia in Barrett’s esophagus

**DOI:** 10.1007/s00259-021-05582-y

**Published:** 2021-12-09

**Authors:** Hsin-Yu Fang, Stefan Stangl, Sabrina Marcazzan, Marcos J. Braz Carvalho, Theresa Baumeister, Akanksha Anand, Julia Strangmann, Julia Slotta Huspenina, Timothy C. Wang, Roland M. Schmid, Marcus Feith, Helmut Friess, Vasilis Ntziachristos, Gabriele Multhoff, Dimitris Gorpas, Michael Quante

**Affiliations:** 1 II Medizinische Klinik, Klinikum Rechts Der Isar, Technische Universität München, Munich, Germany; 2 Department of Radiation Oncology and Central Institute for Translational Cancer Research, (TranslaTUM), Technische Universität München, Munich, Germany; 3 Chair of Biological Imaging, School of Medicine, Technische Universität München, Munich, Germany; Helmholtz Zentrum München, Institute of Biological and Medical Imaging, Neuherberg, Germany; 4 Institute of Pathology, Technische Universität München, Munich, Germany; 5 Department of Medicine, Columbia University Irving Medical Center, New York, NY USA; 6 Chirurgische Klinik, Klinikum Rechts Der Isar, Technische Universität München, Munich, Germany; 7 Innere Medizin II, Universitätsklinik Freiburg, Universität Freiburg, Freiburg im Breisgau, Germany

**Keywords:** Barrett esophagus, Hsp70, Fluorescence molecular endoscopy, Esophageal adenocarcinoma, Surveillance strategies

## Abstract

**Purpose:**

The incidence of esophageal adenocarcinoma (EAC) has been increasing for decades without significant improvements in treatment. Barrett’s esophagus (BE) is best established risk factor for EAC, but current surveillance with random biopsies cannot predict progression to cancer in most BE patients due to the low sensitivity and specificity of high-definition white light endoscopy.

**Methods:**

Here, we evaluated the membrane-bound highly specific Hsp70-specific contrast agent Tumor-Penetrating Peptide (Hsp70-TPP) in guided fluorescence molecular endoscopy biopsy.

**Results:**

Hsp70 was significantly overexpressed as determined by IHC in dysplasia and EAC compared with non-dysplastic BE in patient samples (*n* = 12) and in high-grade dysplastic lesions in a transgenic (L2-IL1b) mouse model of BE. In time-lapse microscopy, Hsp70-TPP was rapidly taken up and internalized  by human BE dysplastic patient–derived organoids. Flexible fluorescence endoscopy of the BE mouse model allowed a specific detection of Hsp70-TPP-Cy5.5 that corresponded closely with the degree of dysplasia but not BE. Ex vivo application of Hsp70-TPP-Cy5.5 to freshly resected whole human EAC specimens revealed a high (> 4) tumor-to-background ratio and a specific detection of previously undetected tumor infiltrations.

**Conclusion:**

In summary, these findings suggest that Hsp70-targeted imaging using fluorescently labeled TPP peptide may improve tumor surveillance in BE patients.

**Supplementary Information:**

The online version contains supplementary material available at 10.1007/s00259-021-05582-y.

## Introduction

The incidence of esophageal adenocarcinoma (EAC) has been rapidly increasing in western countries [1], and Barrett’s esophagus (BE), the metaplastic replacement of the squamous mucosa of the distal esophagus by columnar epithelium, represents the major known precursor lesion for EAC [2]⁠. Similar to EAC, BE incidence has also increased over the past decades, resulting in a large number of individuals “at risk” for EAC. Severe gastroesophageal reflux disease (GERD) is thought to be the primary risk factor for the development of BE. However, BE only develops in up to 10% of patients with GERD, and only a small fraction of patients with BE develops EAC [3–5]⁠, raising questions regarding the utility and best strategy of endoscopic screening.

Histopathological evaluation of endoscopic biopsies has been used to diagnose both dysplasia and cancer, but falls short of sensitive risk prediction, particularly for patients without dysplasia who can still progress to EAC. A more accurate prediction of neoplastic progression would allow for a more focused surveillance of patients at high risk for malignant transformation [6]. Unfortunately, the current endoscopic surveillance strategy has mostly failed, in part because random, non-targeted biopsies often miss significant dysplastic lesions. Studies have suggested that more than 50% EAC cases are missed with this approach [7], pointing to the need for novel imaging tools to better identify potential dysplastic lesions.

We hypothesize that current endoscopic and non-invasive imaging techniques can be improved and enhanced with fluorescence molecular imaging specific for advanced lesions, facilitating the identification of BE patients at increased risk for EAC [8]. Several approaches have recently been studied. For example, fluorescently labeled peptides have been described showing some specificity for dysplasia [7], but have not yet been translated into clinical use [7, 9, 10]. Indeed, improvement in clinical imaging and surveillance strategies for BE have been limited in the past by the absence of tractable pre-clinical models. Several years ago, we developed the L2-IL1b transgenic mouse model of BE that recapitulates the histologic progression to EAC [11]. Indeed, overexpression of IL-1b in these transgenic mice leads over time to chronic esophagitis, Barrett’s metaplasia, and finally EAC in older mice, providing fundamental insights into the pathogenesis of BE [11]. The model has proved useful for testing new preventive and diagnostic strategies.

The search of specific biomarkers being located intracellularly in normal cells, overexpressed in tumor cells, and exposed on the cell surface of malignant cells resulted in the identification of heat shock proteins (Hsps) as promising candidates [12]. We previously demonstrated that the 72 kDa major stress-inducible Hsp70, which is overexpressed in a large variety of highly malignant tumor entities, is also presented on the plasma membrane of those tumors [13]. The tumor-selective cell surface localization of Hsp70 is mediated by a tumor-specific lipid composition which enables the anchorage of Hsp70 in the plasma membrane of tumor cells [14]. The transport of Hsp70 from the cytosol to the plasma membrane is not mediated by a classical ER/Golgi pathway but by a non-classical endo-lysosomal pathway, since inhibitors such as brefeldin A or monensin did not impair the Hsp70 membrane expression on tumor cells [15, 16]. Besides its physiological cytosolic localization in all nucleated normal cells, the major stress-inducible Hsp70 (HspA1A, UniProtKB P0DMV8) is also presented on the plasma membrane of malignantly transformed cells in many different tumor entities. Screening of more than 1200 biopsies of tumor patients has shown that the majority of the primarily diagnosed tumor samples, but none of the corresponding normal tissues, exhibited a membrane Hsp70 (mHsp70)–positive phenotype [17–20]. Upon environmental stress, including standard therapeutic regimens such as radio- or chemotherapy, the synthesis of Hsp70 is induced in the cytosol and the membrane expression density of Hsp70 is increased on therapy-resistant, surviving tumor cells [21]. Therefore, it is expected that mHsp70–targeting tools such as TPP qualify for monitoring of therapy responses. Compared to primary tumors, an upregulated mHsp70 expression is also detectable on relapse tumors and metastases. In multiple studies, it was shown that the malignancy of tumors positively correlates with the Hsp70 expression density on the plasma membrane [18, 22]. Therefore, mHsp70 serves as a biomarker for the prediction of the aggressiveness and metastatic capability of a tumor disease [23].

Since normal cells do not present Hsp70 on their cell surface, mHsp70, in recent years, has emerged as promising tumor-specific target for the in vivo imaging and diagnosis of different malignant lesions [24–29]. Hsp70 is present in most human cell types and appears to play a key role in cancer development, including in EAC [30–32]⁠. Due to its specific membrane expression on tumor cells [27], Hsp70 may represent an ideal target to distinguish malignant from non-malignant tissues. The epitope of the antibody cmHsp70.1 which specifically detects the membrane-bound form of Hsp70 on viable tumor cells is part of the oligomerization domain of Hsp70. Therefore, the 14-mer Tumor-Penetrating Peptide (Hsp70-TPP) derived from the oligomerization domain of Hsp70, covering the epitope of cmHsp70.1 antibody, specifically binds to mHsp70 on tumor cells. Fluorescence- and radionuclide-labeled TPP peptide has been successfully used for tumor-specific in vivo imaging in preclinical models [25, 26]⁠.

Considering the emerging findings supporting a role for Hsp70 in the development of EAC, we investigated the use of fluorescently labeled Hsp70-TPP for in vitro and ex vivo imaging of EAC. We report here a progressive increase in Hsp70 expression from BE to EAC in tissues from L2-IL1b mice and patients, and Hsp70-TPP was taken up rapidly by human organoids in vitro. Systemic or topical administration of Hsp70-TPP-Cy5.5 allowed visualization by fluorescence imaging of dysplastic lesions in both the mouse model of BE and resected human EAC, without expression in non-dysplastic BE. Collectively, our results suggest that Hsp70-targeted fluorescence imaging may be useful to improve early detection of EAC.

## Results

### Hsp70 expression increases with progression of dysplasia in BE in patients and mice

**Fig. 1:**
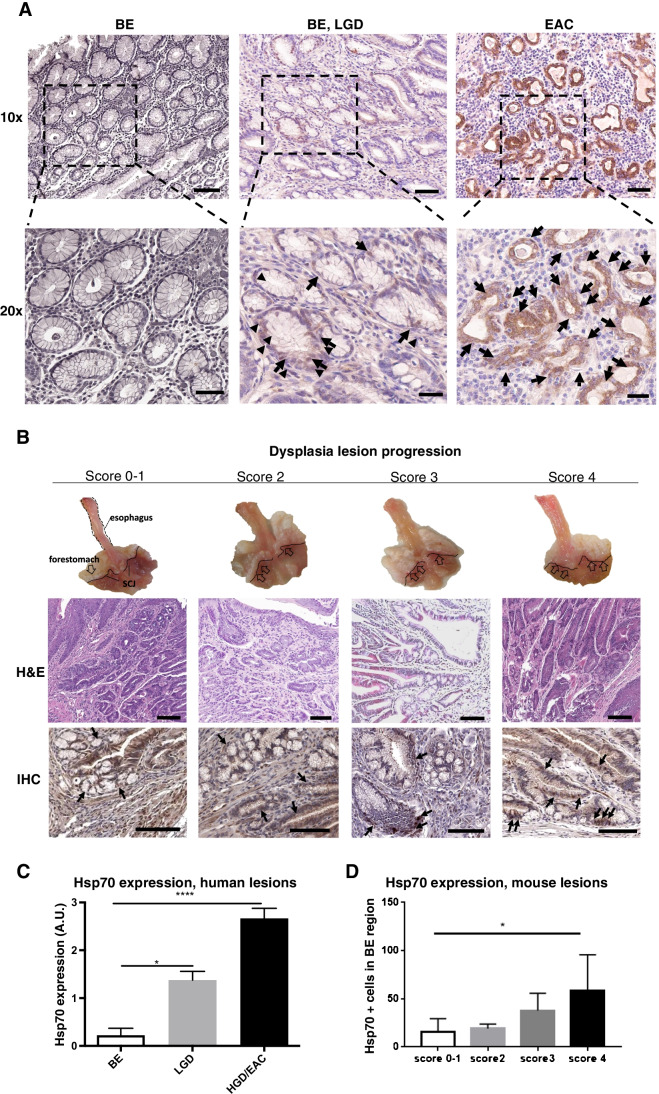
Hsp70 expression increases while dysplasia progresses in high-grade dysplasia (HGD) mice and in EAC patients. (A) Representative images of Hsp70 IHC in patients’ samples: HGD/EAC patients presented a higher Hsp70 expression than low-grade dysplasia (LGD) and BE patients. Most cases of BE showed no expression of Hsp70. Scale bars represent 100 μm (10 ×) and 50 μm (20 ×). (B) Macroscopic picture of esophagus and stomach (open arrow: dysplasia lesions)**.** Representative Hematoxylin & Eosin (H&E) staining of cardia tissue (10 ×). Representative Hsp70 IHC images show that Hsp70 increased through esophageal dysplasia progression (arrows). Brightness and contrast of whole H&E and IHC images were adjusted with ImageJ/Fiji. Scale bars represent 100 μm. (C) Quantification of Hsp70 IHC in 12 patients with BE (*n* = 9), LGD (*n* = 8), and/or HGD/EAC (*n* = 6) assessed using the following semi-quantitative scale: 0—no expression, 1—low expression, 2—moderate expression, and 3—high expression. Data are expressed as mean ± SEM.*P ≤ 0.05 and ****P ≤ 0.0001 by Kruskal–Wallis test. (D) Quantification of Hsp70 IHC in L2-IL1b mice (*n* = 3 ~ 6) assessed on total positive cells in the entire BE region. Hsp70 expression correlated with macroscopic and histopathological scoring. Data are represented as mean ± SEM. *P < 0.05 by one-way analysis of variance (ANOVA)

As recent data indicated that Hsps play a key role in EAC development, we analyzed Hsp70 by immunohistochemistry (IHC) in 12 patients with BE, low-grade dysplasia (LGD), and/or EAC. A significantly higher level of Hsp70 expression was found in human EAC (2.67 ± 0.21 A.U.) as compared to LGD (1.38 ± 0.18 A.U.) and most importantly non-dysplastic BE (0.22 ± 0.15 A.U., Fig. 1A, C). Similarly, we observed strong Hsp70 expression in the transgenic L2-IL1b mouse model, evaluated by counting positive cells in BE region (Fig. 1B). At 9–12 months of age, L2-IL1b mice develop low-grade to high-grade dysplastic lesions in the setting of BE-like metaplasia at the squamocolumnar junction (SCJ), enabling us to study the associated changes [11, 33]. Notably, patients (*p* = 0.0001) and L2-IL1b mice showed a statistically significant increase in the Hsp70 expression during stepwise progression from non-dysplastic BE to LGD to high-grade dysplasia (HGD) and EAC (Fig. 1C and D), suggesting that Hsp70 expression correlates closely with disease progression.

### Human dysplastic BE organoids express Hsp70 and internalize Hsp70-TPP

**Fig. 2:**
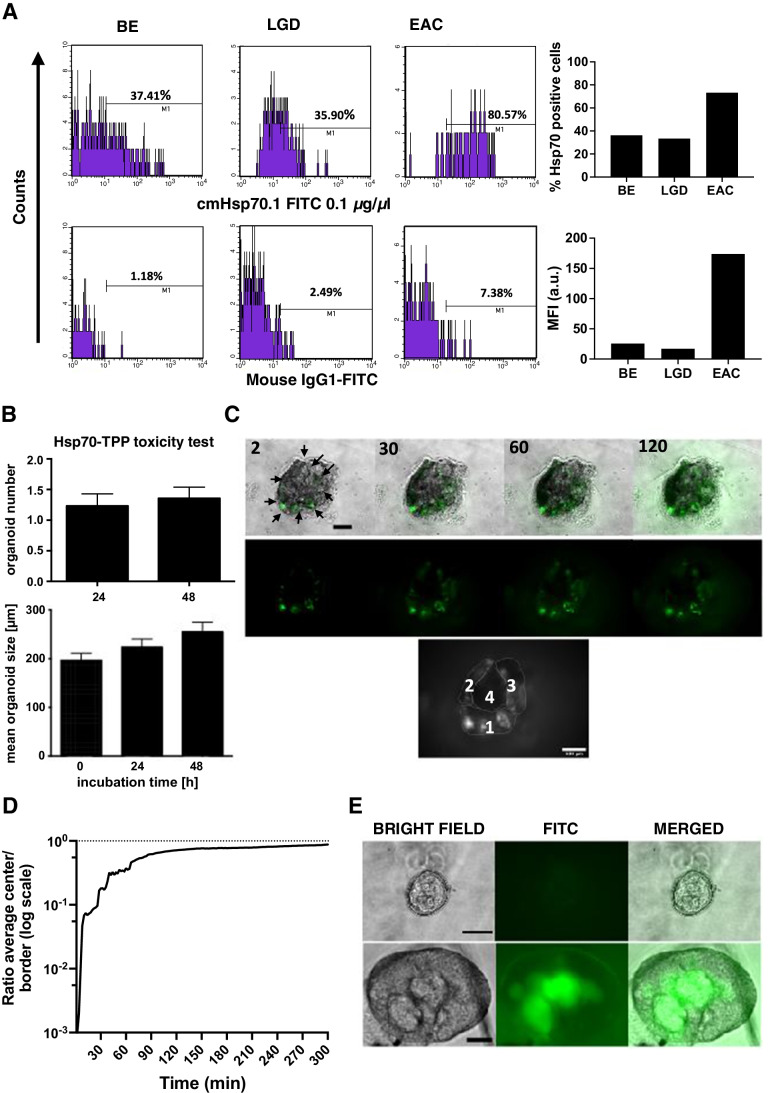
Human organoids present a different expression of Hsp70 and internalize a fluorescent Hsp70-TPP probe. (A) Flow cytometry analysis for Hsp70 expression using anti-Hsp70 antibody (cmHsp70.1 FITC) in human organoids (single cell suspensions from human organoids obtained as described in “Flow cytometry” section) derived from BE without dysplasia (Hu108), LGD (Hu60), and EAC (Hu75). Appropriate isotype controls (Mouse IgG1 FITC) were used for all the analyses. Percentages of positive and viable cells are indicated. Quantification data are expressed as percentage of Hsp70-positive cells and mean fluorescence intensity (MFI). (B) Organoid number at 24 and 48 h after incubation with 200 µg/ml carboxifluorescein-labeled Hsp70-TPP normalized to time 0 (top) and organoid size of BE organoids after treatment (bottom). Data showed no toxicity of Hsp70-TPP for human BE organoids (Hu108, Hu109, Hu115). Data are represented as mean ± SEM. (C) Upper panel: selected frames (bright field/BF + FITC and FITC channel) of time-lapse microscopy of BE organoid (Hu108) after treatment with 100 µg/ml carboxyfluorescein-labeled Hsp70-TPP: time is showed in min. BF and FITC images were acquired every 2 min with automatic exposure time. Scale bar represents 100 µm. Lower panel: organoid ROIs used for the quantification. (D) Quantification of peptide distribution into Hu108 organoid: mean gray values were calculated by making 4 ROIs (C). The intra-organoid signal distribution was then assessed by dividing the average intensity of region 4 by the mean intensity of regions 1–3. Data are represented in log scale. (E) Representative images of human LGD organoids (Hu60) before (above) and after the treatment (below) with 200 µg/ml carboxyfluorescein-labeled Hsp70-TPP. Scale bar represents 50 µm

We next evaluated Hsp70 expression and uptake of Hsp70-TPP in patient-derived 3D organotypic organoids isolated from BE, LGD, or HGD/EAC. In line with the IHC findings, we found an increased expression of mHsp70 in organoids derived from HGD/EAC (> 70%) in comparison with organoids derived from LGD (33%) and BE without dysplasia (36%) (Fig. 2A). Given our in vitro findings suggesting Hsp70 as a potential viable therapeutic target, we investigated potential toxic effects of Hsp70-TPP by treating BE organoids for 48 h with Hsp70-TPP labeled with carboxyfluorescein (CF). No adverse effects on organoid growth or proliferation were detected in these experiments (Fig. 2B).

We then performed time-lapse microscopy of human organoids from a patient with dysplastic BE after treatment with 100 µg/ml Hsp70-TPP-CF (Fig. 2C, Movie S1 A and B). Internalization of Hsp70-TPP in the epithelial cell layer of the organoid could be observed within 10 min after incubation (Fig. 2C, Fig. S1). Quantification of the fluorescence signal in the organoid (Fig. 2D) revealed a progressive signal increase within the organoid after the treatment. We excluded autofluorescence of Matrigel by acquiring pre- and post-treatment images of the FITC channel (Fig. 2E). A fast Hsp70-TPP uptake was also observed in human organoids from LGD after an incubation for 10 min with 200 µg/ml Hsp70-TPP-CF (Fig. S1), consistent with the Hsp70 expression in LGD organoids assessed by flow cytometry. Taken together, these results confirm membrane expression of Hsp70 by dysplastic esophageal cells within organoids and indicate that Hsp70-TPP produce a strong epithelial cell-specific signal without cytotoxicity.

### Fluorescence endoscopy detects dysplastic lesions in mice

**Fig. 3:**
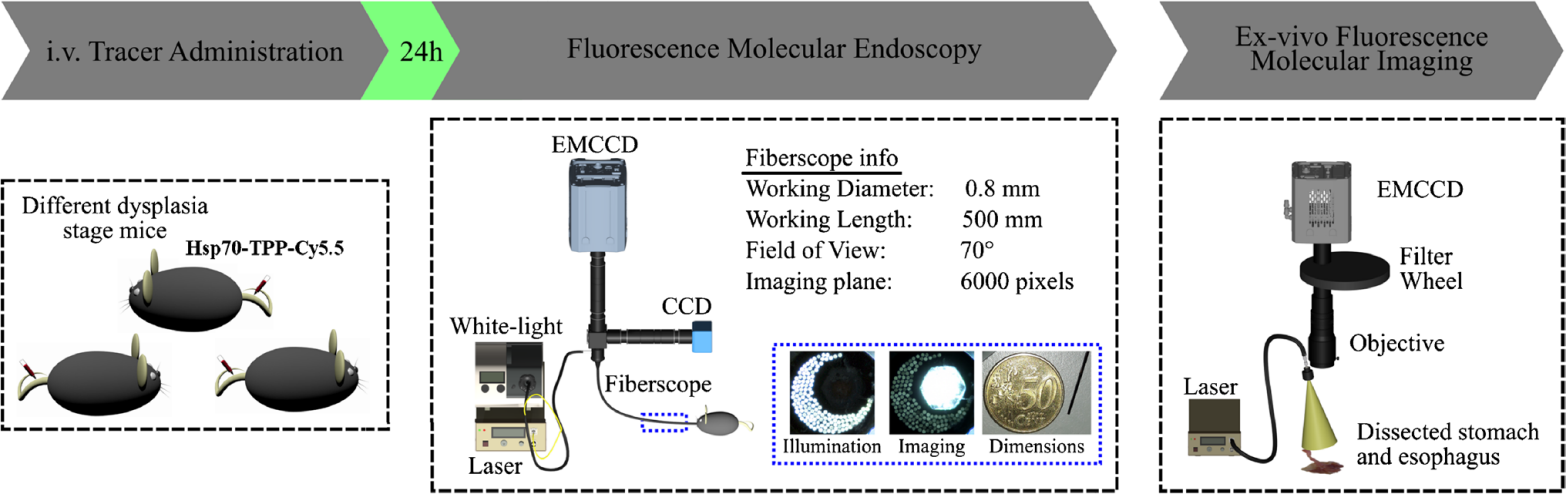
Diagram of experimental procedure. Different age and stages of dysplasia mice were i.v. injected with Hsp70-TPP-Cy5.5. Twenty-four hours post-injection, mice were euthanized for video recording of endoscopy. After that, stomach and esophagus were excised and ex vivo fluorescence imaging was performed. Fluorescence images were acquired by illuminating the specimens using 670 diode laser and guiding the emitted fluorescence through appropriate emission filters before capturing it using a back illuminated EM-CCD camera (iXon DU888, Andor). Legend: CCD, color charge-coupled device; EM-CCD, electron multiplying CCD

**Fig. 4:**
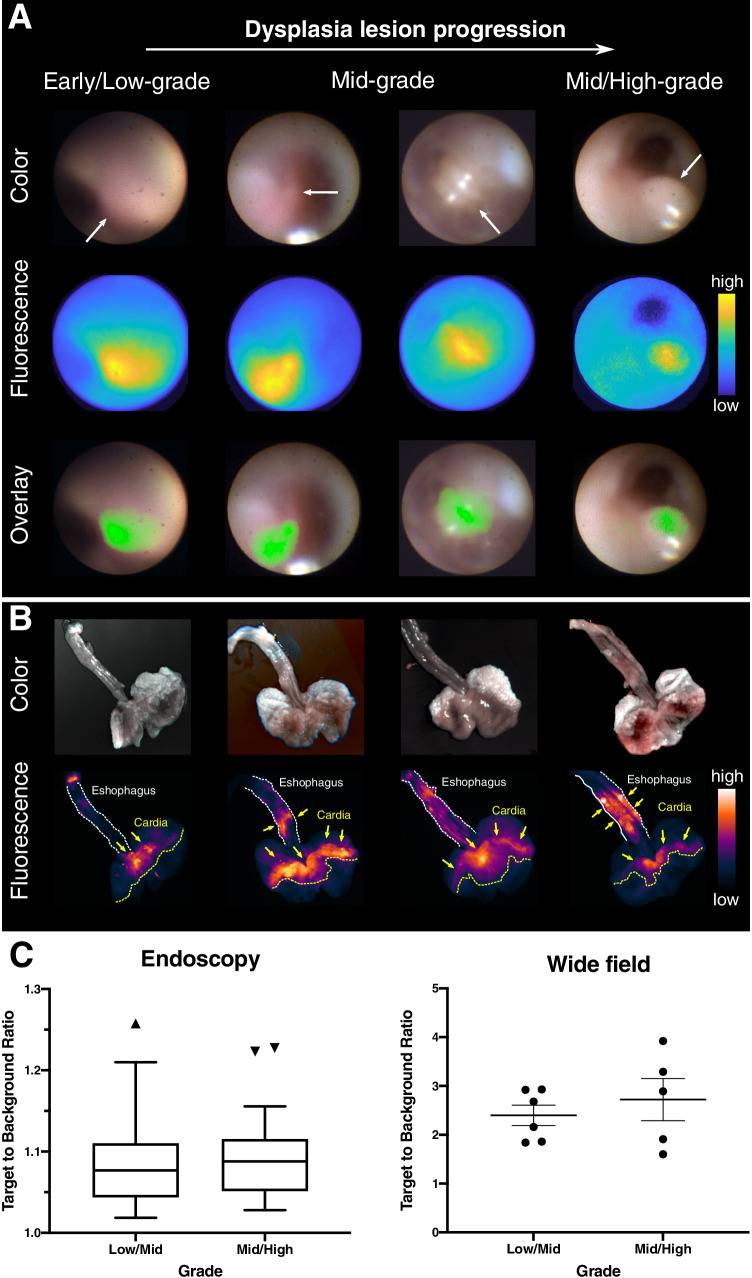
Endoscopy and ex vivo fluorescence imaging show accumulation of Hsp70-targeted fluorescent signal in small dysplasia lesions. (A) Representative white light color (top panel), near-infrared (NIR) (middle panel), and overlay images (bottom panel) from endoscopies performed on L2-IL1b mice with different stages of dysplasia: early/low-grade lesions (6 months old, score 0.50), mid-grade lesions (9 and 12 months old, score 1.50), and mid-/high-grade lesions (9 months old, score 1.75). The scoring of the lesion was performed by averaging the macroscopic and histopathologic dysplasia score of each mouse. In the overlay images, Cy5.5 NIR fluorescence (max ~ 720 nm) is represented in green. (B) Representative fluorescence images of ex vivo imaging showed that uptake of Hsp70 is increased in dysplasia lesions (arrows) of the cardia region (yellow dash line) and esophageal areas in some cases (dash lines). (C) Left: quantification of fluorescence endoscopy in Hsp70-TPP-Cy5.5-injected mice with mid/high-grade lesions (n=5; score 1.75-2.13) shows a higher median target-to-background ratio (TBR) than those with low-/mid-grade lesions (n=6; score 0.5-1.50). Data are represented as box plot, displaying the median and outliers according to Turkey’s method. For each grade, the TBR of 11 to 27 lesions was calculated. Right: quantification of ex vivo Cy5.5 fluorescence signal intensity in the cardia regions (yellow dashline in B; three ROIs per mouse). Signal was quantified with ImageJ by using the ratio of the mean signal intensity and the surrounding background. Data are represented as single plotted values and the mean ± SEM

Based on the findings that local Hsp70 expression increased gradually during esophageal carcinogenesis and with the demonstrated ability of Hsp70-TPP to penetrate organoids, we studied the utility of labeled Hsp70-TPP to diagnose dysplasia in our BE mouse model during endoscopy. Hsp70-TPP-Cy5.5 was injected intravenously (i.v.) into L2-IL1b mice (6, 9, and 12 months old) 24 h prior to post-euthanasia fluorescence upper endoscopy. Fluorescence endoscopy was performed using an in-house custom-made endoscopy system (0.8-mm flexible fiberscope with 6000 pixels imaging plane, see Fig. 3 for other details and Movie S2). Following endoscopy, fluorescence imaging of the excised stomach and esophagus was performed (Fig. 3). Hsp70-targeted fluorescent signal accumulated predominantly in dysplastic lesions at the SCJ (Fig. 4A) and the lesions were also visible in the near-infrared (NIR) channel (Fig. 4A, middle panel). Overlay images (Fig. 4A, bottom panel) showed that Hsp70-TPP-Cy5.5 accumulated in low-, mid-, and mid-/high-grade dysplasia lesions at the SCJ but not metaplastic areas, indicating that fluorescence endoscopy could indeed detect Hsp70 expression even at early dysplastic stage. No NIR signal was detected in a non-injected control IL1b mouse (data not shown). In addition, ex vivo wide-field imaging of the excised stomach and esophagus showed a specific uptake of the Hsp70-TPP-Cy.5.5 specifically in dysplastic lesions of L2-IL1b mice. Of note, the signal intensity was extremely low in the surrounding non-dysplastic epithelium, confirming the specificity of fluorescence endoscopy (Fig. 4B, arrows). Moreover, we observed an increased median target/tumor-to-background ratio (TBR) by endoscopy in injected L2-IL1B mice with mid-/high-grade lesions compared with low-grade lesions (Fig. 4C). However, endoscopy (*p* = 0.6057) and wide-field imaging (*p* = 0.4871) showed no statistically significant difference between the TBR of L2-IL1b mice with mid-/high-grade and early/low-grade lesions by t test (Fig. 4C). Therefore, we conclude that Hsp70-TPP-Cy5.5 can specifically label different stages of dysplastic lesions but not metaplasia or normal tissue in a pre-clinical setting and can be successfully detected using fluorescence molecular endoscopy.

### Imaging of resected human esophagi allows clinical translation of Hsp70-TPP-Cy5.5

**Fig. 5:**
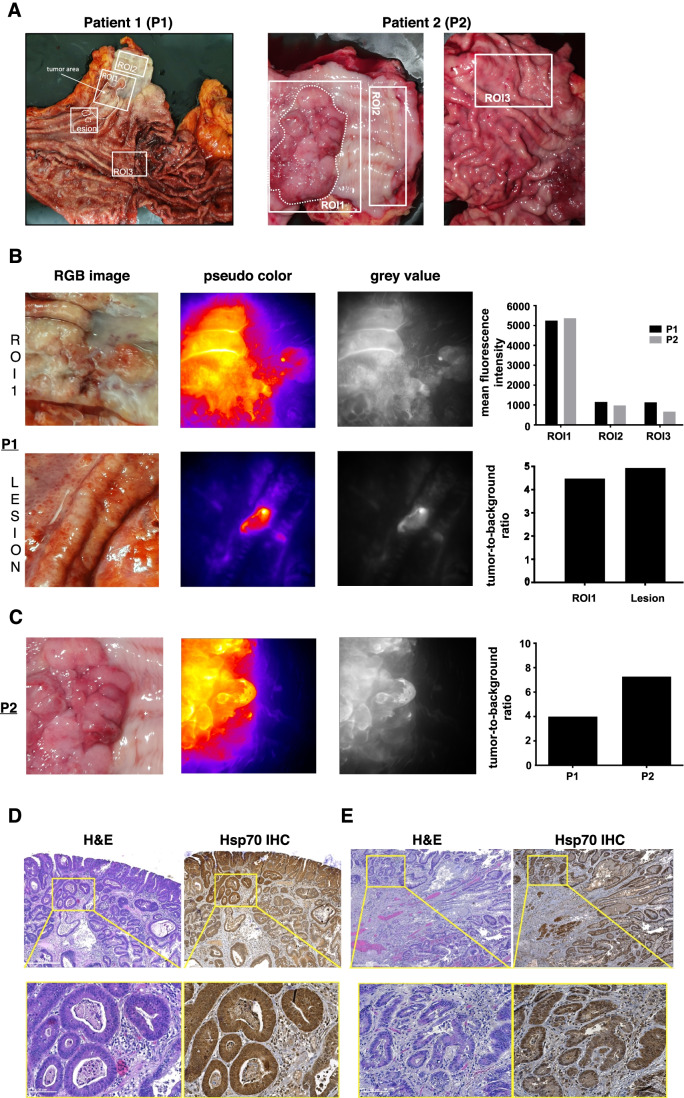
Hsp70-TPP-Cy5.5 spray identifies cancer in excised esophagus of EAC patients (A) Macroscopic color image of the two excised human esophagi: tumor area and the selected ROIs for signal quantification from tumor (ROI1) and macroscopically normal regions of esophagus and stomach (ROI2 and ROI3, respectively) are indicated. (B) Color image, pseudo-color, and gray scale image of ROI 1 and ROI of two additional lesions, patient 1 (left panel). Quantification of the mean fluorescence intensity of tumors and normal tissues from both patients (top right panel). TBR calculated from the tumor area and the two lesions of patient 1 (bottom right panel). TBR was calculated as reported in the “Materials and methods” section. (C) Color image, pseudo-color, and gray scale image of ROI1, patient 2 (left): a high fluorescence signal was detected in correspondence of the tumor after topical application of Hsp70-TPP-Cy5.5 spray. TBR in both human excised esophageal specimens (right). (D) and (E) Histopathological analysis confirmed the presence of esophageal carcinoma and the moderate/high Hsp70 expression in patient 1 (D) and patient 2 (E). Representative images of H&E and Hsp70 IHC of the tumor. Lymphocytes also expressed Hsp70: scale bars represent 500 μm and 100 μm

In order to translate the benefits of Hsp70-specific dysplasia detection into the clinical setting, we investigated the performance of the labeled Hsp70-TPP peptide applied topically to primary human specimens. In preparation to this experiment, we validated the binding of Hsp70-TPP in a xenograft subcutaneous mouse model. In this model, specific binding of Hsp70-TPP-Cy5.5 to the exposed tumor cells was observed following topical administration of the peptide (Fig. S2A). The mean penetration depth of the peptide-tracer, as measured on 1-mm slices from the tumors, was 0.52 ± 0.05 mm (Figure S2) after 5 min of application. Next, Hsp70-TPP-Cy5.5 was applied topically to primary resected human esophageal specimens from two EAC patients undergoing esophagectomy without prior chemotherapy. Hsp70-TPP-Cy5.5 was topically applied to the resected fresh tissue, prior to formalin fixation, including tumor (ROI1, Fig. 5A, B upper panel, and C) and non-tumor areas (ROI2 and ROI3, Fig. 5A). After 5 min of incubation, the quantified fluorescence signal (Fig. 5B, upper panel, right) showed a higher fluorescence intensity in tumor areas (ROI1) compared with non-tumorigenic esophagus (ROI2) and stomach (ROI3). Of note, adjacent BE tissue did not show any signal, but two additional previously unrecognized lesions were identified in a macroscopically normal appearing area of the resected specimen of patient 1 (P1) after Hsp70-TPP-Cy5.5 application (Fig. 5A and B, lower panel). The lesions showed a high and specific TBR, underlining the potential for improved fluorescence-guided biopsy protocols (Fig. 5B, lower panel, right). In addition, a high TBR (≥ 4) was observed in the tumor areas of both patients (Fig. 5C, right). Finally, histopathological analysis of all esophageal specimens confirmed the diagnosis of EAC with expression of Hsp70 by IHC (Fig. 5D and E). Importantly, non-dysplastic BE regions of the excised esophageal samples did not show any signal, providing evidence of high specificity of Hsp70-TPP-Cy5.5 for the detection of dysplasia and EAC.

## Discussion

In this study, we provide pre-clinical and translational evidence for the utility of the Hsp70-targeting peptide TPP as a specific molecular marker to detect early dysplastic esophageal lesions. We analyzed the expression of Hsp70 in patient-derived organoids and primary resection specimens, with minimal or no expression in non-dysplastic BE and increasing expression with increasing dysplastic grades, from LGD to HGD and EAC. The important role of Hsp70 as a marker for early dysplasia was further confirmed in our L2-IL1b mouse model, which provides further evidence for a stepwise increase of Hsp70 during progressive dysplasia development. Before translating our results to humans, we analyzed the tracer of interest in a pre-clinical model. Indeed, in the BE mouse model with a novel fluorescence molecular endoscopy system, Hsp70 was specifically detected in early and late esophageal dysplastic lesions. Importantly, the specificity of Hsp70-TPP-Cy5.5 for early dysplasia and EAC was then confirmed using ex vivo human samples, providing proof-of-concept for this approach.

In the last decade, targeted endoscopic imaging has emerged as a promising strategy to overcome the significant number of EACs missed in BE surveillance using conventional white-light endoscopy [7]. To date, several studies have reported the clinical use of fluorescent peptides for dysplasia detection in patients with HGD or EAC [9, 10, 34]. Sturm et al. used the ASY*-FITC peptide with endoscopy first in a pilot study of 25 patients [9], followed by a larger second one [34], but despite high specificity for HGD and EAC (94%), the sensitivity of ASY*-FITC was only 76% [34]. In another study, fluorescence endoscopy using Alexa Fluor 680 was used to detect glycosylation patterns associated with HGD and EAC in human biopsies and resected esophagi [35], but this approach has not yet been transferred into clinical use [35]. Other studies have reported in vivo EAC detection after administration of labeled antibodies to epidermal growth factor receptor 2 (HER2) [36] and fibroblast growth factor receptor 2 (FGFR2) [37]. Nevertheless, these approaches have yet to be confirmed in large clinical studies.

Previously, we have explored the use of human hybrid fluorescence molecular endoscopy in combination with white light endoscopy in BE patients, demonstrating that topical Bevacizumab-IRDye800CW administration has potential to improve BE surveillance strategies [38, 39]. Although promising in selecting at-risk patients, VEGF targeting alone might lack specificity in larger cohort of BE patients. Additionally, we have reported on the use of imaging the tumor microenvironment using CXCR4-targeted PET/CT imaging in EAC [40]. Here, we investigated the value of the tumor cell–specific marker Hsp70. We used the NIR fluorophore Cy5.5, which presents a low tissue autofluorescence, high penetration depth, photostability, and high specificity [37], leading to a TBR of ≃1.1 by mouse endoscopy and an impressive TBR (≥ 4) after topical application on human esophageal specimens. The difference in TBR between L2-IL1b mice and human esophageal specimens may relate to the strong inflammatory background along with the lesser degrees of dysplasia in the BE mouse model or to lower Hsp70 expression in mouse tissues (see Figs. 4 and 5). In addition, we employed different administration protocols (i.e., topical for human and systemic for mouse tissues). Nevertheless, while we did not observe a statistically significant difference between TBR of early and late stages of dysplasia by mouse endoscopy and wide-field imaging, the data together support the usefulness of Hsp70-TPP-Cy5.5 in guiding biopsies to detect dysplasia.

Despite contradictory results on the expression of Hsps in the esophageal epithelium [41], Hsp70 has emerged as a prognostic biomarker in EAC, with high expression associated with aggressive behavior [30], higher chemosensitivity [31]⁠, and with higher tumor grade and poor prognosis, confirmed in a more recent study on patients surgically treated for EAC [32]. This study also showed no Hsp70 expression in normal esophagus and BE, but an increase in dysplastic lesions, correlating with Hsp70 expression observed in esophagogastric junction adenocarcinoma but not in the normal adjacent tissue [42]. Together with our previous studies showing the utility of Hsp70-TPP-Cy5.5 for cancer imaging [26, 43], our results provide evidence of a possible clinical translation of Hsp70-TPP-Cy5.5.

Additionally, our study highlights the utility of testing biomarkers in relevant animal models prior to studying such markers and/or fluorescent peptides in human samples. Prior to the introduction of the L2-IL-1b mouse model, this has been quite difficult in the esophageal cancer field. Indeed, the use of integrin αvβ3/CD51-targeted epirubicin-loaded RGB NIR self-assembled peptide nanoparticles (RGB-f-PNPs; diameter: 30 nm) was recently reported in a subcutaneous xenograft model of EAC for theranostic purposes [44], but the absence of non-dysplastic BE in the model is a notable limitation. Preclinical models that harbor BE, LGD, HGD, and EAC, such as the L2-IL-1b model, are likely necessary in order to assess cancer development in the appropriate preneoplastic setting [45]. In addition, the use of a highly biocompatible compound with a low production cost, an easy design, and the conjugation with highly biocompatible or already clinically approved dyes are other factors that may accelerate clinical translation. Compared with other agents tested for cancer imaging like antibodies [36] and nanoparticles [44, 46]⁠, peptides such as Hsp70-TPP offer several advantages such as smaller size, lower production costs, lower immunogenicity and toxicity, higher tumor penetration, and faster body clearance [26, 37].

Within this study, we modified a robust endoscopic system that provides real-time co-registered fluorescence and color information, developed earlier by our group [47] to be suitable for mouse endoscopy. The use of a miniaturized flexible fiberscope (0.8-mm diameter) enables performance of upper endoscopic imaging on small animals. Moreover, we designed the system to allow for imaging different fluorescent dyes by exchangeable filters within the fluorescence detection optical path, thus making the endoscope a unique tool for the pre-clinical investigation of BE and EAC biomarkers.

Although the findings of the study are highly promising, we acknowledge that we investigated Hsp70 expression only in a small number of patients and a few excised esophageal samples. More information is needed regarding Hsp70 expression in a larger cohort of patients with dysplasia and EAC and confirmation of these preliminary data by more detailed studies in the future is necessary. Furthermore, the specificity of the NIR signal from Hsp70-TPP-Cy5.5 and the use of fluorescence molecular endoscopy were tested in one pre-clinical model of BE (L2-IL1b). While this model consistently progresses from BE to dysplasia/EAC [11, 48], there are important differences between mice and humans that need to be taken in account [49]⁠.

In summary, we demonstrated the pre-clinical and possible clinical utility of an Hsp70-targeting fluorescently labeled peptide Hsp70-TPP for specific and early detection of dysplasia or EAC. The fluorescence molecular endoscopy system allowed specific detection of fluorescent NIR peptides, paving a way for future applicability of such system in clinical studies. However, further studies are needed to establish the potential role of this Hsp70 imaging modality in human BE surveillance and EAC screening.

## Materials and methods

### Study design

The objective of the present study was to evaluate the feasibility of using Hsp70 as a predictive marker of EAC by ex vivo fluorescence molecular endoscopy in mouse models of BE and ex vivo imaging on human tissues. The correlation of Hsp70 expression with EAC progression was first evaluated by immunohistochemistry (IHC) on dysplasia and EAC tissues from 12 patients and IHC Hsp70 data were then compared with our L2-IL1b mouse model of BE at different time points (6, 9, and 12 months; *n* = 3–6 per group). We then evaluated Hsp70 expression in vitro using patient-derived organotypic BE cultures with different degree of dysplasia (*n* = 3/4) by flow cytometry and treated them with the Hsp70-specific 14-mer Tumor-Penetrating Peptide (Hsp70-TPP) labeled with carboxyfluorescein/CF. After treatment, we used time-lapse microscopy on organoids culture with different degree of dysplasia (*n* = 2) to visualize peptide internalization. In addition, we evaluated the growth and proliferation of BE organoids isolated from 3 different patients in duplicates before and after 24 and 48 h of treatment with Hsp70-TPP. Experiments were then performed using our mouse model of BE L2-IL1b and endoscopic evaluation for Hsp70 was performed 24 h after the injection of Hsp70-TPP labeled with Cy5.5 by a custom-made fluorescence molecular endoscopy system. One non-injected mouse (L2-IL1b mouse, female, 12 months old) served as control. The endoscopy was performed using 6-, 9-, and 12-month-old male and female mice, with a sample size of 2–3 to 6 per age group.

The specificity of the Hsp70-TPP-Cy5.5 signal detected by endoscopy was further confirmed by performing wide-field imaging on the excised stomach and esophagus and the fluorescence signal was quantified in a blind fashion, without knowing the mice score. The number of animals analyzed is indicated in the figure legends. Finally, preliminary data of topical application of Cy5.5-labeled Hsp70-TPP for EAC detection were obtained using a xenograft mouse model of pancreatic carcinoma and excised esophageal specimens from patients with EAC (*n* = 2) without prior chemotherapy. Fluorescence from Cy5.5 was measured in the tumor region and normal regions (at least 2 background ROIs per sample) and TBR was calculated using mean intensity values. Blinding was not used.

### Three-dimensional organoid culture

3D organoids were isolated from esophageal biopsies of patients with BE, dysplasia, or HGD/EAC using a slightly modified protocol [50]⁠. Briefly, the tissue was cut in 1–2-mm pieces and incubated with Accutase (ThermoFisher) for 20 min at RT with gentle shaking. After the incubation, EDTA-PBS without calcium and magnesium (DPBS, 2 mM) was added followed by PBS plus 10% FBS and 1% penicillin–streptomycin (PS) and the sample was centrifuged at 500 rcf for 7 min at 4 °C. After centrifugation, the pellet was resuspended in 50 μl of Matrigel (Corning no. 356231) and seeded in a 24-well plate with L-WRN-derived media plus growth factor supplements. Human organoids derived from BE without dysplasia (Hu108, Hu115, and Hu109), BE with dysplasia (Hu60), or EAC (Hu75) were then maintained at 37 °C in a humidified atmosphere of 5% CO_2_ and 95% air and media was changed every 2–3 days. Experiments were then performed before 6 passages.

### Tumor-penetrating peptide (Hsp70-TPP) and cytotoxicity evaluation

Hsp70-TPP peptide was found to specifically bind membrane-bound as well as cytosolic residing Hsp70 by mimicking the proteins’ oligomerization domain, mediating its in vivo tumor specificity in many different solid tumors [27]. Previous toxicity studies on murine tumor models revealed no toxic side effects of Hsp70-TPP peptide [26, 27]. In the present study, we investigated for the first time the cytotoxicity of Hsp70-TPP-CF on human tissues. BE organoids (*n* = 3; passage 3–6) were incubated for 48 h with 200 µg/ml of the peptide added to the culture media. For this experiment, the organoid viability and proliferation were assessed before and after an incubation time of 24 and 48 h by measuring size and number of viable organoids. Experiments were performed in duplicate. To measure the diameter of the organoids, pictures (5–10 images per organoid type per each time point) were taken with an inverted microscope (Zeiss Axiovert 200 M) and the size of each organoid was measured with ImageJ/Fiji software [51], using the mean of 3 diameters. The average size of all three organoid types was then calculated at each time point by using the mean size of 11 to 27 organoids for each organoid type. Data are represented as the mean ± SEM. The number of organoids for each type counted at 24 and 48 h after incubation was normalized by dividing it to the organoid count at time 0. Data are represented as the mean of normalized data from all three organoid types ± SEM.

### Time-lapse microscopy

For the time-lapse microscopy, organoids (passage 3–6) were incubated with cell recovery solution (Corning) for 10 min on ice, in order to dissolve the embedding Matrigel. PBS plus 10% FBS was then added to each well and the whole content was placed in a Falcon tube on ice for 30 min. After the Matrigel was dissolved, the organoids were washed and subsequently incubated with Hsp70-TPP-CF (100–200 μg/ml diluted in PBS). After an incubation of 10 min and washing, the pelleted organoids were resuspended in 50 μl of Matrigel and seeded in a 2-well or 4-well chamber slide (Lab-Tek II Chamber Slide). Time-lapse microscopy was then started immediately and performed over a course of 1–8 h using an epifluorescence inverted microscope (Zeiss AxioObserver Z1). Images were acquired in bright field and FITC channel every 60–120 s with automatic exposure time. Time-lapse videos were then processed and brightness and contrast of the whole images were further adjusted using ImageJ/Fiji software.

### Flow cytometry

To confirm the expression of membrane-associated Hsp70 in malignant cells of the human organoids, flow cytometry was performed on single cell suspensions from human organoids as follows. Untreated, viable organoids (passage 3–6) were washed with PBS plus 10% FBS and subsequently disassembled by incubation in EDTA-trypsin (Sigma-Aldrich, St. Louis, MO, USA), supplemented with 0.5U/ml collagenase (Roche Diagnostics, Mannheim, Germany) for 10 min at 37 °C. After enzyme deactivation with PBS plus 10% FBS, the single cell suspension from organoids was centrifuged again and the pellet was incubated with isotype-matched (IgG1) control antibody (BD Biosciences) or FITC-conjugated cmHsp70.1 antibody for 30 min at 4 °C protected from the light as described previously [43]⁠. Only viable cells (propidium-iodine negative) were then analyzed using a FACSCalibur flow cytometer (BD Biosciences, Franklin Lakes, NJ, USA). Flow cytometry data were then quantified by using the mean fluorescence intensity and/or the percentage of Hsp70-positive cells, obtained after subtracting the contribution from cells bound to the IgG1 control antibody.

### Animals

All animal experiments were performed with protocols approved by the District Government of Bavaria and in accordance with the German Animal Welfare and Ethical Guidelines of the Klinikum rechts der Isar, TUM (Munich, Germany). All procedures were performed in accordance with the National Institutes of Health Guide for the Care and Use of Laboratory Animals. Genetic mouse models over-expressing IL1b (L2-IL1b) were generated as previously reported and backcrossed to C57BL/6 J mice [11, 33]⁠. Mice were fed with water and standard chow diet (Ssniff, V1124-000) ad libitum and were genotyped between 6 and 8 weeks of age. For the endoscopy experiments, L2-IL1b mice of 3 different age groups (6, 9, and 12 months; *n* = 2/3–6 per group) were used.

### Histology

For histology, L2-IL1B mice of 3 different age groups ( 6, 9, and 12 months) representing disease progression were sacrificed by isoflurane overdose . The esophagus and stomach of L2-IL1B mice were resected, formalin-fixed (10%), paraffin-embedded, and sectioned. Slides were then processed for H&E staining. Histological scoring was performed by an experienced mouse pathologist using a blinded scoring system, using previously established criteria for the influx of immune cells per high-power field, metaplasia, and dysplasia in mice [11, 33]. Inflammation represents a score of all immune cells within a defined area of tissue around the squamocolumnar junction (SCJ), predominantly made up of neutrophil myeloid cells. Metaplasia was assessed through identification of mucous-producing cells per gland, and the number of glands with mucous-producing cells in the BE area. Dysplasia was assessed by evaluating cellular atypia in the presence of low- and high-grade dysplasia within each gland. For final scoring, the histological scoring of each mouse was averaged with the macroscopic scoring of the lesions, performed by evaluating tumor extension into the cardia and tumor size as reported by Munch et al. [33].

For human tissues, BE, LGD, and HGD/EAC tissues from esophagectomy or endoscopic resection specimens from 12 patients were fixed in formalin, embedded in paraffin blocks, and stained for H&E and Hsp70 IHC. Images were then acquired with an Aperio Slide Scanner (Leica Biosystems, Wetzlar, Germany).

### Immunohistochemistry (IHC)

After deparaffinization and rehydrating to water, FFPE tissue Sects. (2 µm) from patients and L2-IL1B mice of 3 different age groups (6, 9, and 12 months; *n* = 3–6 per group) were heated by microwaving for 30 min in pH6 target retrieval buffer (Agilent DAKO, Santa Clara, CA, USA) to unmask antibody epitopes. Non-specific binding was blocked by protein blocking solution (5% v/v rabbit serum/antibody diluent (REAL antibody diluent, Agilent DAKO, Santa Clara, CA, USA)). Sections were then washed in PBS (Sigma-Aldrich, St. Louis, USA) after each step. For Hsp70 IHC, sections were incubated overnight at 4 °C with the mouse monoclonal antibody cmHsp70.1 at a concentration of 2 µg/ml (multimmune GmbH, Munich, Germany), followed by HRP-labeled anti-mouse secondary reagent (Agilent DAKO, Santa Clara, CA, USA). Staining was visualized with 3,3-diaminobenzidine (DAB1) chromogen (Agilent DAKO, Santa Clara, CA, USA) for 4 min, consistent for all staining procedures. Nuclei were counterstained with hematoxylin. Then, sections were embedded in Eukitt (Sigma cat# 03,989) mounting medium. Appropriate quality control and quality assurance procedures were implemented including positive (FaDu) and negative (surrounding tissue) control tissues run with each assay.

Quantification of Hsp70 was performed in the mouse samples by counting the Hsp70 + cells (brown) in the entire BE region. In human samples, the expression of Hsp70 was semi quantitatively assessed in the cells of the whole BE, dysplasia, and/or EAC region by an experienced gastroenterologist as the following: 0—no expression, 1—low expression, 2—moderate expression, and 3—high expression.

### Fluorescence molecular endoscopy

The high sensitivity fluorescence/color endoscope that was built for this project is based on an imaging system developed by our group [47]. Specifically, the imaging setup was designed to offer video-rate simultaneous color and near-infrared (NIR) fluorescence endoscopy. The system employs a multipurpose flexible endoscope for small animal imaging (i.e., 0.8-mm outer diameter, 6000 pixels, Micrendo-Fiberskop, SCHÖLLY FIBEROPTIC GMBH, Denzlingen, Germany). White light illumination for color imaging is provided by a 250-W halogen lamp (KL-2500 LCD, Schott AG, Mainz, Germany) while the fluorescence excitation is provided by a fiber-coupled continuous wave (CW) laser diode emitting at 670 nm (SLD1332V, Thorlabs, Newton, NJ, USA). The power at the distal end of the endoscope complies with the American National Standards Institute (ANSI) and the European Standards (EN) limits for the maximum permissive exposure in skin (40 mW/cm^2^ measured at distance < 2 mm). Both light sources are coupled into a multimode bifurcated fiber-bundle (Leoni FiberOptics, Neuhaus-Schierschnitz, Germany) connected to the light guide of the fiberscope. The acquired optical signal is divided by a beam splitter (FF685-Di02, Semrock, Rochester, NY, USA) into a visible and a NIR channel. The visible channel is recorded by a high-resolution color charge-coupled device (CCD) camera (pixelfly qe, PCO AG, Kelheim, Germany), through relay lenses (MAP10100100-A, Thorlabs), while a highly sensitive electron-multiplying CCD (EMCCD) is used for the detection of the NIR channel (DV897DCS-BV, Andor Technology, Belfast, Northern Ireland). In order to enable the use of the system with various fluorophores, a dichroic mirror rotating holder has been installed (CDFW5/M, Thorlabs), while sliding holders (CFS1/M, Thorlabs) allow for the interchangeability of the filters so that optimal imaging is achieved under any fluorophore.

For fluorescence endoscopy, male and female L2-IL1b mice (7 males, 4 females) with different grades of dysplasia were used. Mice were i.v. injected with Hsp70-TPP-Cy5.5 (OEM manufactured by Thermo Fisher; dosage: 100 µg, equal to 45 nmol per animal) and sacrificed 24 h post-injection through anesthetic overdose. Immediately after the sacrifice, endoscopy was then performed by advancing the endoscope into the esophagus while air was gradually introduced to allow esophagus distension. Videos were recorded and stored in AVI file format. The TBR from the fluorescence endoscopy images was quantified by averaging the pixel intensity values within two ROIs (tumor and background ROI) and taking their ratio. The selection of the ROIs was performed by defining the fluorescence signal from BE and macroscopically visible dysplastic lesions and the surrounding normal esophageal mucosa was defined as the background.

### Ex vivo* wide-field fluorescence molecular imaging*

To verify the in vivo biding capabilities of Hsp70-TPP-Cy5.5 to target malignant lesions and to validate the fluorescence molecular endoscopy, we used an ex vivo fluorescence imaging system to detect Hsp70-TPP-Cy5.5-derived fluorescence signals on exposed or excised specimen. For near-infrared epifluorescence imaging in tissue, specimens were illuminated using a 670-nm diode laser (B&W tek, DE, USA). The emitted fluorescence was guided through a 780/10 bandpass filter and captured with a back-illuminated EM-CCD camera (iXon DU888, Andor), as described previously [27]. Fluorescence images were sequentially acquired at exposure times of 0.2, 0.5, 1, and 2 s. Comparative analysis has been carried out at images taken with identical exposure times, objective apertures, and camera settings. Signal specificity was determined by calculating the ratio of the mean signal intensities of the relevant tissue and the adjacent normal tissue using ImageJ as previously reported [52]. Data are represented as single plotted values and/or mean ± SEM. Imaging procedures of the different types of specimen have been performed as follows.

### *Epifluorescence *in vivo* Hsp70 imaging of mouse tumor tissues*

To verify the applicability of Hsp70-TPP-Cy5.5 to specifically label malignant tissue using topical application, a subcutaneous (s.c.) xenograft human pancreas carcinoma (Colo357) model and the IL-1b overexpressing genetic (*L2-IL1b*) mouse model were used. Animals with xenograft tumors were euthanized when s.c. tumors reached a volume of 0.2 to 0.3 cm^3^ and tumors and adjacent tissues were excised. L2-IL1b mice received an endoscopic imaging after sacrificing them and the whole esophagus and stomach were spread flatly on a black coated aluminium foil before imaging. Xenograft s.c. tumors were spread flatly on a black coated aluminum foil and incubated with an aerosol of Hsp70-TPP-Cy5.5 at a concentration of 200 µg/ml in PBS, for 5 min at room temperature. After extensive rinsing of the tissue with PBS, fluorescence images from the exposed areas were taken, as described above. To quantify the penetration depth of Hsp70-TPP-Cy5.5 in malignancies, fresh s.c. tumors were subsequently cut transversally and longitudinally into 1-mm-thick slices directly after exposure to the Hsp70-TPP-Cy5.5 aerosol. Fluorescence imaging was performed, as described above.

### *Epifluorescence *in vivo* Hsp70 imaging of human esophageal specimens*

For fluorescence imaging on human specimens, resected fresh human esophageal carcinomas and normal tissues were collected from two patients with EAC undergoing curative esophagectomy without prior chemotherapy. After collection, excised tissues were rinsed in PBS before topical spray application of Hsp70-TPP-Cy5.5 at a concentration of 200 µg/ml to the total area of the specimen (~ 420 cm^2^). After incubation for 5 min at RT, the specimens were extensively rinsed in PBS and imaged as described above. The specific binding of Hsp70-TPP-Cy5.5 was quantified with ImageJ by using the ratio between the mean fluorescence intensity of the tumor area and the mean fluorescence intensity in the normal tissue. After imaging, the tissues were fixed in 10% formalin, embedded in paraffin blocks, and sections were stained for H&E and Hsp70. IHC for Hsp70 was performed as reported in “Immunohistochemistry (IHC)” section.

### Statistical analysis

Quantitative data were analyzed by one-way analysis of variance (ANOVA) followed by *post hoc* Tukey test, two tailed unpaired t test (quantification of endoscopy) and  nested t test (quantification of ex vivo wide-field imaging) . Statistical analysis of semi-quantitative data was performed by Kruskall-Vallis test and differences in the means were evaluated using Dunn’s multiple comparison test. All statistical analyses were performed with GraphPad Prism Software (San Diego, CA, USA) and data are represented as single plotted values and/or mean ± SEM. *P* ≤ 0.05 was considered significant.

## Data and material availability

Data related to this study can be found in the paper or supplementary materials.

## Supplementary Information

Below is the link to the electronic supplementary material.

Supplementary file1 (DOCX 15.9 MB)

Supplementary file2 (MOV 14161 KB)

Supplementary file3 (MOV 13970 KB)

Supplementary file4 (AVI 63496 KB)
