# Supplementary material for: Targeted Hsp70 fluorescence molecular endoscopy detects dysplasia in Barrett’s esophagus
Source: Eur J Nucl Med Mol Imaging. 2021 Dec 9;49(6):2049–63. doi: 10.1007/s00259-021-05582-y (PMC9016004; doi:10.1007/s00259-021-05582-y)
Supplement: Supplementary file 1 — Supplementary file1 (DOCX 15.9 MB) [file 259_2021_5582_MOESM1_ESM.docx]

**Supplementary Materials:**

**Fig. S1. Uptake of Hsp70-TPP peptide occurred in human BE and LGD organoids.** (**A**) Representative image in bright field/BF, FITC, and merged channel of another Hu108 organoid after 10 min incubation with 100 µg/ml carboxyfluorescein-labelled Hsp70-TPP (Hsp70-TPP-CF). The fluorescence signal localized in the epithelial cells of the organoid. Scale bar represents 100 µm. **(B)** Representative time-lapse microscopy images of Hu60 organoid after 10 min incubation with Hsp70-TPP-CF (200 µg/ml) followed by washing as reported in the Methods sections: selected frames in BF, FITC and merged channel showed peptide distribution into the epithelial cell layer of Hu60 organoid (arrows). Time-lapse microscopy (automatic exposure time; 1 min frame interval) was started after 5 min at 37°C to allow Matrigel solidification. Scale bar represents 100 µm. **(C)** Quantification of intra-organoid distribution of fluorescence signal shows signal increase (up to 0.9-1.0 ratio) within the whole organoid: measured organoid ROIs (left) and signal quantification (right) after the start of time-lapse. The intra-organoid distribution was assessed by calculating the ratio between the average intensity of regions 2 or 3 and region 1. Data are reported in linear scale.

**Fig. S2. Validation of Hsp70-TPP-Cy5.5 topical administration (A)** Fluorescence image of s.c. xenograft model of pancreatic tumor after topical application of Hsp70-TPP-Cy5.5 as spray (pseudo-colored). The tumor was then cut in half, displaying the tagged outer shell and non-stained tumor volume. **(B)** Hsp70 IHC of the tumor showed strong Hsp70 expression in the tumor cells. **(C)** Black and white image of a 1mm slice of the tumor. **(D)** Pseudo-colored fluorescence image of the slice shown in **(C)** showing Hsp70-TPP-Cy5.5 accumulation in the outer layer of the tumor. **(E)** histogram of the gray values along the line depicted in the inlay (α to ω). The mean penetration depth of the peptide-based tracer was 0.52 ± 0.05 mm. The measurement was taken at the locations of the biggest change in gray value.

**Movie S1.** **Time-lapse microscopy of human BE organoid in Matrigel (0-7.5 hours)**. Progressive internalization of Hsp70-TPP-CF is shown in BF and FITC channel (Movie S1 A) and merged (Movie S1 B) are shown. Videos resolution is 1080p with a speed of 15 fps.

**Movie S2**. **Endoscopy video of a dysplasia BE mouse.** Representative endoscopy video in a L2-IL1b mouse with mid-grade lesions 24 hours after Hsp70-TPP-Cy5.5 injection: images acquired by the high-resolution color CCD camera (left), highly sensitive fluorescence camera (middle) and merged video (right). The NIR signal is colored in yellow and in green in the fluorescence and merged video, respectively, and it co-localized with macroscopically visible lesions. Videos’ resolution is 720p.
